# Activation and Regulation of Cellular Eicosanoid Biosynthesis

**DOI:** 10.1100/tsw.2007.180

**Published:** 2007-09-01

**Authors:** Thomas G. Brock, Marc Peters-Golden

**Affiliations:** Division of Pulmonary and Critical Care Medicine, Department of Internal Medicine, University of Michigan Health System, Ann Arbor, MI, USA

**Keywords:** eicosanoid, biosynthesis, regulation, arachidonic acid, cyclooxygenase, prostanoids, 5-lipoxygenase, leukotriene, lipoxin

## Abstract

There is a growing appreciation for the wide variety of physiological responses that are regulated by lipid messengers. One particular group of lipid messengers, the eicosanoids, plays a central role in regulating immune and inflammatory responses in a receptor-mediated fashion. These mediators are related in that they are all derived from one polyunsaturated fatty acid, arachidonic acid. However, the various eicosanoids are synthesized by a wide variety of cell types by distinct enzymatic pathways, and have diverse roles in immunity and inflammation. In this review, the major pathways involved in the synthesis of eicosanoids, as well as key points of regulation, are presented.

## INTRODUCTION

Eicosanoids (from the Greek *eikosi* for "twenty") are a family of oxygenated metabolites of the 20-carbon fatty acid arachidonic acid (AA) that are released from the source cell and act at nanomolar concentrations on target cells, typically via G-protein coupled receptors (GPCRs). Although originally recognized for their capacities to elicit biological responses such as smooth muscle contraction, edema, and platelet aggregation, eicosanoids are now appreciated to influence processes ranging from inflammation and immune responses to chronic tissue remodeling and cancer. Indeed, there is scarcely an area of biology or medicine in which these lipid mediators have not been implicated. Moreover, a variety of pharmacotherapeutic strategies based on eicosanoids are in current use and others are on the horizon. This paper will provide a general overview of eicosanoid biosynthesis with emphasis on prostanoids (prostaglandins[PGs] and thromboxane), leukotrienes (LTs), and lipoxins.

## OVERVIEW OF BIOSYNTHETIC PATHWAYS

The synthesis of eicosanoids (Fig. 1) depends on the availability of free AA for subsequent metabolism. Such availability, in turn, is influenced by the relative proportion of AA among the unsaturated fatty acids esterified at the *sn*-2 position of membrane phospholipids, as well as its relative capacities for enzymatic removal from (deacylation) and reinsertion into (reacylation) phospholipids. The AA content of phospholipids can vary

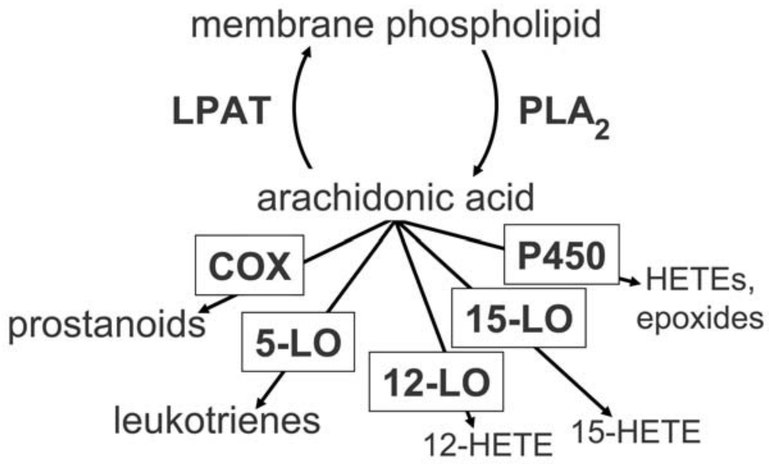

**FIGURE 1.** Overview of eicosanoid synthesis. AA is released from membrane phospholipid by $PLA_2$ enzymes and may be reacylated into phospholipid by lysophospholipid acyltransferase (LPAT). Free AA is metabolized by different enzymes, including COX, 5-LO, 12-LO, 15-LO, and cytochrome P450, to produce the different eicosanoids.

among different cell types. It is also influenced by dietary intake because increased consumption of n-3 fatty acids, which are enriched in fish oils, such as eicosapentaenoic and docosahexaenoic acids, tends to result in decreased AA content and a corresponding decrease in synthesis of bioactive eicosanoids. Deacylation is catalyzed by the enzyme phospholipase $A_2$ ($PLA_2$)[1]. This process is stimulated by cellular agonists (see below), acting via the generation of second messengers. More than 15 different mammalian $PLA_2$ enzymes have been identified, which differ in their intracellular localization and biochemical characteristics; the major families are cytosolic, secretory, and calcium-independent $PLA_2$s. It is likely that each of these families contributes to eicosanoid synthesis in particular cells and situations, and that interactions between members of different families are essential for optimal fatty acid hydrolysis under certain circumstances. Cytosolic $PLA_2$-alpha (c$PLA_2$-α) appears to be the most universally important in mediating the stimulated synthesis of eicosanoids. This is an AA-preferring enzyme whose activation involves its calcium-dependent translocation from the cytosol to intracellular membranes. Reacylation of fatty acids back into phospholipids is catalyzed by acyl-CoA:lysophospholipid acyltransferase, and activity of this enzyme can likewise be regulated.

Free AA is potentially available for bioconversion to eicosanoids via a variety of metabolic pathways, each generally referred to by the name of its initial oxygenase enzyme. The two pathways about which the most information exists are the PGH synthase or cyclooxygenase (COX) pathway and the 5-lipoxygenase (5-LO) pathways; each will be discussed at greater length below. In both of these, the oxygenase catalyzes the synthesis of an unstable intermediate, which is further metabolized by downstream terminal enzymes. Others include the 12- and 15-LO as well as the cytochrome P450 oxygenation pathways, giving rise to 12- and 15-hydroxyeicosatetraenoic acids (HETEs) and epoxyeicosatrienoic acids, respectively.

## REGULATION OF EICOSANOID GENERATION

Consistent with their biological importance, the synthesis of eicosanoids is carefully regulated (Table 1). The most obvious mechanism for regulation is via changes in expression of their biosynthetic enzymes. Expression of virtually all of the proteins involved in lipid mediator synthesis is subject to regulation, either up or down. Changes in expression most often involve regulation at the transcriptional level. Factors modulating expression of eicosanoid-forming proteins include cytokines, hormones, vitamins, and

**TABLE 1**
**Mechanisms for the Regulation of Eicosanoid Biosynthesis and Secretion**

| |
|---|
| Alteration in expression levels of biosynthetic proteins |
| Genetic |
| Acquired/induced (modulation by cytokines, hormones, lipopolysaccharide, etc.) |
| Alterations in function of biosynthetic proteins |
| Levels of essential cofactors (e.g., calcium, ATP, glutathione) for enzymes |
| Post-translational modifications that influence catalytic activity of proteins (e.g., phosphorylation, nitration) |
| Intracellular localization of proteins |
| Alteration in expression or function of transport proteins required for export |

microbial products. In addition to such exogenous modifiers, genetic factors can also influence the expression of many of these proteins, as polymorphisms in the promoter or coding elements of their corresponding genes have been identified.

The catalytic efficiency of eicosanoid-forming enzymes is also subject to regulation. This can occur through a variety of mechanisms. First, levels of essential cofactors for these enzymes can vary. For example, ATP is required for optimal 5-LO action, and reduced glutathione is required for optimal action of PGE synthase and as a cosubstrate for synthesis of $LTC_4$; depletion of ATP or glutathione, as might occur during metabolic or oxidative stress, can thereby influence the profile of eicosanoids generated. Second, post-translational modification of enzymes can influence their catalytic efficiency; examples include phosphorylation by protein kinases and nitration by nitric oxide. In this way, mitogen-activated protein (MAP) kinases can enhance the actions of $cPLA_2$, protein kinase A can reduce the actions of 5-LO, and nitric oxide can directly inhibit 5-LO. Third, intracellular compartmentalization of eicosanoid-forming proteins has emerged as an important means of regulating their functions. As lipid mediators are predominantly secreted into the extracellular space, it was long assumed that their synthesis depended on phospholipid hydrolysis and metabolism at the plasma membrane. Unexpectedly, many of the enzymes involved in their generation have instead been localized to the nuclear membrane and perinuclear endoplasmic reticulum in activated cells. This is the case for $cPLA_2$-$\alpha$ as well as oxygenases and terminal synthases of both COX and 5-LO pathways. It may be inferred that topographic colocalization of a group of enzymes that must function in series serves to optimize their coupling and the efficiency for lipid mediator generation. As colocalization of enzymes may depend on specific molecular motifs or phosphorylation events, it too may be subject to genetic or acquired variation.

Finally, the fact that lipid mediators are synthesized within the cellular interior mandates that mechanisms for their export must exist. Indeed, evidence suggests the presence of distinct cellular transporters for prostanoids, $LTB_4$, and $LTC_4$. Although much remains to be clarified about these export mechanisms, they represent an additional target for variation and regulation.

## ACTIVATORS OF EICOSANOID SYNTHESIS

A myriad of substances can stimulate or potentiate the synthesis of lipid mediators. Three types of stimuli are recognized.

1. “Complete activators” (see Table 2) are capable of rapidly triggering AA release and its subsequent metabolism to an array of eicosanoids, often because they increase intracellular calcium either directly or via receptor-dependent signal transduction. Examples include the model activator calcium ionophore as well as physiologically relevant substances, such as hormones, microbes, antigens, and oxidants.

**TABLE 2**
**Representative "Complete" Activators of Eicosanoid Biosynthesis**

| | |
|---|---|
| Soluble pharmacological agents | Calcium ionophores |
| | Phorbol esters |
| | Thapsigargin |
| Receptor-mediated agonists | Formyl peptide (fMLP) |
| | C5a complement fragment |
| | Thrombin |
| | Bradykinin |
| | Interleukin-8 |
| | Endothelin |
| | Immune complexes |
| | Leukotrienes |
| | Platelet activating factor (PAF) |
| Microorganisms | Viruses |
| | Bacteria |
| | Fungi |
| | Protozoa |
| Phagocytic particles | Zymosan |
| | Asbestos |
| | Silica |
| | Certain microorganisms |
| Oxidants | Hyperoxia |
| | Ozone |
| | Nitrogen dioxide |

2. Certain agonists are only weak triggers by themselves, but can potentiate the subsequent response to a more complete agonist in a process referred to as "priming". A priming effect may reflect activation of protein kinases that amplify the catalytic activity or translocation of eicosanoid-forming enzymes. Lipopolysaccharide and various cytokines act in this manner.
3. Finally, a number of substances can potentiate lipid mediator synthesis by up-regulating expression of eicosanoid-forming enzymes, as noted above; cytokines exemplify such actions. The effects of a given agonist on lipid mediator generation may vary depending on the cell type.

## CELLULAR PARTICIPATION IN EICOSANOID SYNTHESIS

Although virtually all cell types participate in eicosanoid synthesis, the specific pathways expressed and, therefore, the spectrum of products synthesized, depend on the cell type. As a general rule, structural or parenchymal cell types (such as epithelial cells, endothelial cells, fibroblasts, and smooth muscle cells) metabolize AA primarily via the COX pathway, often with some modest capacity for 12- or 15-LO metabolism. By contrast, only bone marrow–derived cells tend to synthesize large amounts of 5-LO products, and some also generate substantial amounts of COX, 12-LO, or 15-LO metabolites.

The specific profile of eicosanoids synthesized by a given cell type is dictated by its complement of distal enzymes (see Table 3). However, it is instructive to note that both tissue- and species-specificity in eicosanoid synthetic profiles can also be manifest. For example, alveolar macrophages tend to synthesize substantially more LTs and less prostanoids than do macrophages from other sites. Furthermore, mouse alveolar macrophages differ from human or rat cells in that $LTC_4$, rather than $LTB_4$, is their predominant 5-LO product. In view of the differences in biological actions between these two LTs, this species discrepancy must be kept in mind when seeking to interpret the human significance of studies employing transgenic mouse models in which macrophages are a key source of these mediators.

**TABLE 3**
**Eicosanoid Synthetic Profiles of Various Cell Types**

| Cell Type | $TxA_2$ | $PGI_2$ | $PGD_2$ | $PGE_2$ | cysLT | $LTB_4$ |
|---|---|---|---|---|---|---|
| Platelet | +++ | | | | | |
| Neutrophil | +/– | | | +/– | | +++ |
| Alveolar macrophage | + | | +/– | + | + | +++ |
| Mast cell | | | +++ | | +++ | + |
| Endothelial cell | | +++ | | + | | |
| Epithelial cell | | + | | +++ | | |
| Smooth muscle cell | | +/– | | +++ | | |
| Fibroblast | | +/– | | +++ | | |

*In vivo*, lipid mediator synthesis reflects interactions among neighboring cells. In a process known as transcellular eicosanoid synthesis, one cell type releases an intermediate in the AA metabolic pathway that is subsequently taken up and processed to an eicosanoid by a second cell type. Thus, studies in a variety of coculture systems have documented transcellular metabolism of intermediates including AA itself, $PGH_2$, and $LTA_4$ to classical eicosanoids, such as prostanoids and LTs. Furthermore, transcellular exchange of LO intermediates can result in dual oxygenation of AA by both 5-LO and either 12- or 15-LO, to yield lipoxygenase interaction products, or lipoxins (see below). Such transcellular interactions can involve virtually any combination of bone marrow–derived and structural cell types, and are clearly important determinants of tissue output of lipid mediators[2].

## PROSTANOID SYNTHESIS: THE CYCLOOXYGENASE PATHWAY

Prostanoids comprise a family of lipid mediators that includes $PGE_2$, $PGD_2$, and $PGF_{2\alpha}$, as well as prostacyclin ($PGI_2$) and thromboxane $A_2$ ($TxA_2$). Prostanoids have long been known to modulate such biological processes as smooth muscle tone, vascular permeability, pain, fever, and platelet aggregation. More recently, roles for these molecules in cellular proliferation and survival, immune responses, and even sleep have been identified. The clinical importance of prostanoids is emphasized by the fact that their biosynthesis is the target for nonsteroidal anti-inflammatory drugs (NSAIDs), one of the most widely used classes of pharmacotherapeutic agents.

AA is transformed to the unstable endoperoxide $PGH_2$ by the COX enzymes (Fig. 2). Since the early 1990s, it has been recognized that COX exists in two isoforms, generally designated COX-1 and COX-2. Most cell types are capable of expressing both COX isoforms, and both can initiate prostanoid synthesis from AA. The initial paradigm held that COX-1 was constitutively expressed in most tissues and was responsible for basal synthesis of prostanoids that mediated “housekeeping” functions, while COX-2 expression required induction (by cytokines, hormones, growth factors, lipopolysaccharide), which was glucocorticoid-inhibitable, and was responsible for augmented synthesis of prostanoids that mediated pathobiologic effects. From this came the notion that inhibition of COX-2 accounted for the desirable therapeutic effects of nonselective NSAIDs (e.g., aspirin, indomethacin, and ibuprofen) that inhibited both isoforms, while inhibition of COX-1 accounted for their unwanted side effects (e.g., gastrointestinal irritation and impaired renal blood flow). This paradigm promptly ascended to the level of dogma, and helped to drive the swift development of highly selective COX-2 inhibitors (coxibs). In fact, only some of

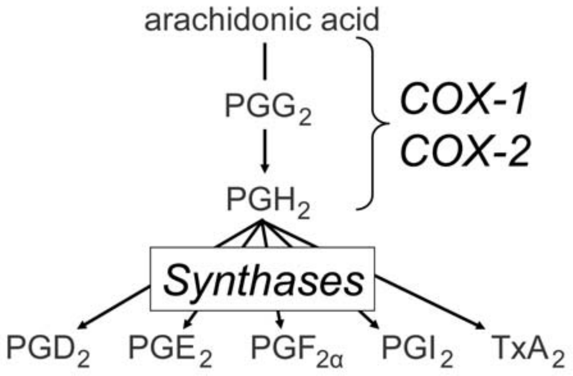

**FIGURE 2.** The cyclooxygenase pathway. The enzymes COX-1 and COX-2 initiate the synthesis of prostanoids from AA, producing $PGH_2$ in a two-step process. The generation of end-products from $PGH_2$ is mediated by specific synthases that are distributed in a cell-specific manner.

the predictions of the original dogma have proven to be accurate. The prediction that synthesis of pathologic prostanoids most often derives from COX-2 has generally been borne out. Not anticipated, however, was the fact that COX-2 was expressed constitutively in certain tissues (e.g., the vascular endothelium and the respiratory epithelium) and was consequently responsible for elaboration of prostanoids serving certain homeostatic functions.

Despite the fact that clear differences in regulation and tissue distribution of the two COX isoforms exist, they do coexist in a number of cells and tissues. This has raised the question of whether they might subserve different functions. Indeed, some evidence suggests that they might preferentially contribute to the synthesis of distinct prostanoids. For example, studies in primary peritoneal macrophages, which naturally express all of the possible terminal PG synthases, suggested that COX-1 yielded a balance of prostanoids while COX-2 preferentially generated $PGE_2$ and $PGI_2$[3]. *In vivo* differences in isoform-specific prostanoid output were recently confirmed in transgenic mice in which COX-1 was “knocked in” under the control of the COX-2 promoter[4]. Work in heterologous expression systems has suggested specific patterns of coupling between either COX-1 or COX-2, and specific upstream $PLA_2$s and downstream PG synthases. Localization of these COX isoforms to distinct intracellular membranes could potentially explain such results and it has been reported in some experimental systems that COX-2 has a greater propensity to be associated with the nuclear membrane while COX-1 distribution is more likely confined to the endoplasmic reticulum[5]; however, such differences have not been found consistently[6].

The COX-derived intermediate $PGH_2$ is converted to bioactive prostanoids by terminal synthase enzymes, whose expression profiles exhibit cell type specificity. Although macrophages synthesize a broad range of prostanoids, most cell types elaborate a single predominant COX product, as follows: platelets, $TxA_2$; mast cells, $PGD_2$; endothelial cells, $PGI_2$; and epithelial cells, fibroblasts, and smooth muscle cells, $PGE_2$. $PGF_{2\alpha}$, $PGI_2$, and $TxA_2$ synthases appear to represent single enzyme isoforms. Lipocalin and hematopoietic PGD synthases represent two distinct gene products present in brain and mast cells/macrophages, respectively. At least three PGE synthases exist, all of which require reduced glutathione as a cofactor. Cytosolic PGE synthase and membrane-associated PGE synthase-2 are both primarily constitutively expressed enzymes. Membrane-associated PGE synthase-1, by contrast, resembles COX-2 in its induction by inflammatory stimuli, which can be inhibited by glucocorticoids.

Ambient prostanoid levels in tissues depend not only on their rate of synthesis, but of degradation as well. It has long been known that during a single pass through the pulmonary vascular bed, the majority of prostanoids are inactivated. This occurs via transporter-mediated uptake into endothelial cells followed by metabolism via the enzyme 15-hydroxy PG dehydrogenase. However, it is increasingly recognized that expression of this dehydrogenase also influences local prostanoid levels and actions, and mice in which this enzyme has been deleted demonstrate enhanced levels of and biological responses mediated by $PGE_2$. Expression of this enzyme too can be modulated by cytokines.

## LEUKOTRIENE SYNTHESIS: THE 5-LIPOXYGENASE PATHWAY

The LTs are produced primarily by inflammatory cells like polymorphonuclear leukocytes, macrophages, and mast cells, and, therefore, have central roles in inflammatory responses. $LTB_4$ attracts and activates neutrophils, monocytes, and lymphocytes, contributing to the increase in activated leukocytes that is a hallmark of tissue inflammation. The cysteinyl LTs ($LTC_4$, $LTD_4$, and $LTE_4$) cause plasma leakage from postcapillary venules, leading to the edema that is also characteristic of inflammation. $LTD_4$ and 5-oxo-6,8,11,14-eicosatetraenoic acid (5-oxo-ETE) are eosinophil chemoattractants. Each of the 5-LO products has a distinct role in promoting different aspects of inflammation.

The biosynthesis of LTs from AA is, on the surface, a simple two-step process (Fig. 3). Synthesis is initiated by the enzyme 5-LO, activated by the 5-LO activating protein (FLAP), and completed by one of two downstream enzymes. For the production of $LTB_4$, the second enzyme is $LTA_4$ hydrolase, whereas $LTC_4$ synthase catalyzes the production of $LTC_4$. As the expression of 5-LO is largely restricted to leukocytes, the biosynthesis of LTs, for the most part, requires these cells.

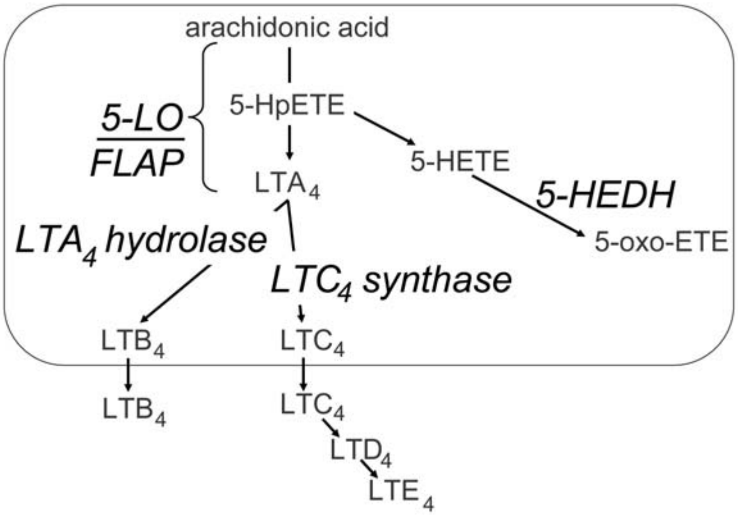

**FIGURE 3.** The 5-LO pathway. The synthesis of LTs from AA is initiated by 5-LO and FLAP, with $LTA_4$ hydrolase and $LTC_4$ synthase completing the production of $LTB_4$ and $LTC_4$, respectively. The by-product 5-HETE can be converted to 5-oxo-ETE by 5-HEDH.

Unlike the COX enzymes, 5-LO is typically inactive in resting cells. As a result, PGs, but not LTs, may be produced whenever free AA is available. 5-LO is activated by an increase in intracellular calcium[7], and this activity can be enhanced by ATP[8]. 5-LO may also be activated by phosphorylation[9], and this activation can occur without an increase in calcium[10]. In resting cells, 5-LO is soluble, but on cell activation, 5-LO associates with intracellular membranes, which presumably places it close to FLAP and free AA. Surprisingly, the substrate AA itself facilitates the association of 5-LO with nuclear membranes[11].

The action of 5-LO actually involves two sequential catalytic reactions: first, inserting molecular oxygen at the fifth carbon of AA to produce 5-hydroperoxyeicosatetraenoic acid (5-HpETE), followed by its dehydration to the unstable $LTA_4$. The intermediate 5-HpETE, often made in significant amounts, is rapidly modified to 5-HETE. $LTB_4$ is produced from $LTA_4$ by the insertion of a hydroxyl group at the 12 carbon, whereas $LTC_4$ synthesis involves linking the tripeptide glutathione at the sixth carbon via the cysteine residue of glutathione. $LTB_4$, and presumably 5-HETE, is exported from the cell by an unidentified protein carrier[12]. $LTC_4$ is exported by the ATP-dependent multidrug resistance proteins[13], including MRP1 and MRP2. After export, $LTC_4$ is readily metabolized by cleavage of the gamma glutamyl portion of glutathione to produce $LTD_4$, and this is further modified by peptidase-mediated removal of glycine to give $LTE_4$.

An additional product whose synthesis is initiated by 5-LO activity is 5-oxo-ETE. This eicosanoid can be produced enzymatically by the oxidation of 5-HETE by 5-hydroxyeicosanoid dehydrogenase (5-HEDH), which is found in leukocytes and platelets[14]. Alternatively, 5-oxo-ETE can be produced nonenzymatically from other lipid mediators[15,16]. This product has potent biological activities that are only now being appreciated. Furthermore, 5-oxo-ETE can be further modified by both enzymatic and nonenzymatic pathways to produce several additional eicosanoids[17]. 5-Oxo-ETE and its derivatives are not classically considered to be LTs.

Early studies on the regulation of LT synthesis assumed that pathway enzymes functioned at or near the plasma membrane, since the products are secreted. This simplifying assumption led to a focus on the role of gene expression in regulating LT synthesis. Most commonly, slow changes, on the order of days, occur in the expression of many enzymes of the 5-LO pathway, typically accompanying leukocyte differentiation and maturation[18,19,20,21]. More rapid changes have been reported in neutrophils, where GM-CSF can increase the expression and translation of 5-LO and FLAP over hours[22,23]. However, rapid, significant changes in gene expression of 5-LO pathway enzymes are not typically seen.

In the 1990s, researchers began to realize that many of the enzymes involved in AA metabolism were at or near the nuclear envelope. For example, FLAP was found to be embedded in the nuclear envelope and endoplasmic reticulum[24]. The key enzyme 5-LO, which is soluble in its resting form, was found to be imported from the cytoplasm to the nucleus in a regulated fashion[25,26,27]. This import of 5-LO into the nucleus is distinct from the membrane association described above, as it occurs without activation of the enzyme and does not result in LT synthesis. The subcellular localization of 5-LO affects $LTB_4$ synthesis: 5-LO produces more $LTB_4$ when positioned within the nucleus than when localized in the cytoplasm[25,28]. The enzyme $LTA_4$ hydrolase can also move into the nucleus of macrophages and rat basophilic leukemia cells, but apparently not in neutrophils[29]; the colocalization of $LTA_4$ hydrolase with 5-LO may serve to enhance the conversion of $LTA_4$ to $LTB_4$. Curiously, nuclear localization of 5-LO in eosinophils serves to strongly decrease the amount of $LTC_4$ produced on cell activation[30].

The 5-LO pathway is also modulated by phosphorylation. 5-LO is phosphorylated on Ser523 by protein kinase A, and this both reduces enzymatic activity[31] and leads to accumulation of 5-LO within the cytoplasm[32]. 5-LO can also be phosphorylated, *in vitro*, on Ser271 by MAP kinase-activated protein kinase 2 and calmodulin kinase II[9,33], and on Ser663 by ERK2[34], although phosphorylation does not alter enzyme activity *in vitro* and has yet to be demonstrated to occur in cells. Phorbol esters can inhibit $LTB_4$ synthesis and may be able to trigger phosphorylation of $LTA_4$ hydrolase[35]. Alternatively, $LTA_4$ hydrolase appears to be constitutively phosphorylated on Ser415 and inactive in human endothelial cells[36]. Finally, phorbol esters may phosphorylate $LTC_4$ synthase[37], but the effect of phosphorylation on activity is unknown. These findings suggest that phosphorylation may be an important way to modulate the 5-LO pathway, although much remains to be elucidated.

## LIPOXIN BIOSYNTHESIS

Lipoxins are lipid mediators that have important roles in the resolution of inflammation[38]. They inhibit neutrophil adhesion, chemotaxis, degranulation, and superoxide generation[39,40,41,42] and stimulate monocyte adherence and chemotaxis without causing degranulation or release of reactive species[43,44]. Structurally, lipoxins are products of AA metabolism that have three hydroxyl groups. The enzymatic pathways that lead to the formation of the lipoxins are thought to involve the interaction of two different cell types. For example, the production of lipoxins A4 ($LXA_4$) and B4 ($LXB_4$) may involve, first, the synthesis of $LTA_4$ by the 5-LO pathway in neutrophils, followed by the conversion of $LTA_4$ to lipoxins by 12-LO in adjacent platelets (Fig. 4A). Alternatively, lipoxin synthesis may involve the synthesis of 15(S)-HpETE by 15-LO in epithelial cells, followed by the conversion of 15(S)-HpETE to the intermediate 15(S)-epoxytetraene by 5-LO in adjacent leukocytes (Fig. 4B). The intermediate may then be hydrated extracellularly to produce the lipoxins. Lastly, the so-called "aspirin-triggered" lipoxins may be produced following the modification of COX-2 by acetylation by aspirin (Fig. 4C). While acetylated COX-2 cannot produce PGs, it can still generate 15(R)-HpETE. This product can then be further processed by the 5-LO pathway in neighboring leukocytes to produce 15(R)-epoxytetraene, giving rise to 15 epi-$LXA_4$ and 15 epi-$LXB_4$.

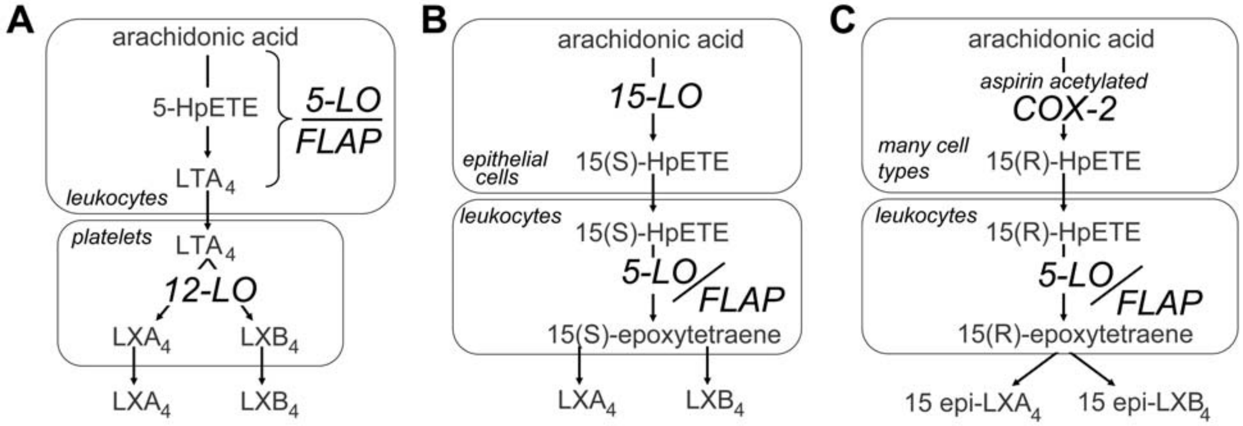

**FIGURE 4.** Biosynthesis of lipoxins. Lipoxins can be produced during cell-cell interactions, utilizing enzymes in adjacent cells. Either $LXA_4$ or $LXB_4$ can be synthesized from AA by either (A) a 5-LO/FLAP to 12-LO pathway or (B) a 15-LO to 5-LO/FLAP sequence. (C) Aspirin-triggered lipoxins are sequential action of aspirin acetylated COX-2 and 5-LO/FLAP on AA.

Levels of lipoxin generation would be expected to depend on the activity of the requisite interacting lipoxygenases. While this has been observed for 15-LO[45], results are conflicting for 5-LO. Thus, 5-LO knockout mice exhibited lower levels of lipoxin generation *in vivo*[46], yet patients with cystic fibrosis[47] and severe asthma[48], conditions recognized to manifest intense LT overproduction, were reported to be *deficient* in $LXA_4$ generation. These results emphasize our incomplete understanding of the determinants of lipoxin synthesis.

Clearly, lipoxin synthesis will be dependent on the proximity of the cells that are necessary for the transcellular metabolism of AA to the lipoxin end products. Also, lipoxin synthesis will depend on the expression, post-translational modification, and subcellular distribution of the pathway enzymes. As 5-LO and FLAP are essential for lipoxin synthesis, many of the mechanisms that impact LT synthesis may also affect lipoxin synthesis. For example, phosphorylation of 5-LO on Ser523 should reduce metabolism of 15-HpETE and lipoxin synthesis, much as it reduces metabolism of AA to LTs. Other processes that modulate 5-LO and FLAP function, including subcellular distribution and colocalization, should also impact lipoxin synthesis.

Relevant to lipoxin synthesis involving 15-LO, there are also two types of 15-LO, referred to as 15-LOX-1 and 15-LOX-2, and both are found in leukocytes and epithelial cells, as well as other cell types[49]. While 15-LOX-1 appears to be restricted to the cytoplasm[50,51], 15-LOX-2 may be imported into the nucleus[51,52]. As both isoforms produce the same product and can occur in the same cell, this strongly suggests that the movement of one isoform, but not the other, serves to alter the subcellular synthesis of 15-HpETE. It is possible that this redistribution of 15-LOX-2 in epithelial cells may alter the amount of secreted product, thus changing the amount of substrate that is available for lipoxin synthesis by neighboring leukocytes. Alternatively, the nuclear import of 15-LOX-2 in leukocytes might alter its positioning relative to 5-LO in the same cell, reducing or enhancing lipoxin synthesis within a single cell.

It is interesting that the classical platelet-type 12-LO, which has been shown to produce lipoxins, is broadly distributed and can be found in epithelial cells[53,54], smooth muscle cells[55], and leukocytes[56]. This 12(S)-LO is overexpressed in a variety of tumors including breast, prostate, pancreatic, and renal cancer[57] and in psoriasis[54]. This suggests that lipoxins may be formed by a variety of cell types from $LTA_4$ produced by adjacent leukocytes, and overproduced in some diseases. Also, a 12(R)-LO exists in epithelial cells, but whether it can function in lipoxin generation is unknown.

## FUTURE DIRECTIONS

In this limited space, only the major eicosanoids could be introduced and only key features regarding their synthesis and the regulation of their production could be presented. While this underscores the diversity of this group of mediators, it must be stressed that our understanding on this topic is far from complete. Undoubtedly, exciting discoveries regarding eicosanoid biosynthesis and its regulation lie ahead. Perhaps even more exciting will be future discoveries on the roles of eicosanoids in health and disease, and the way that these findings can be utilized for treatment.

## ACKNOWLEDGMENTS

This work was supported by National Institutes of Health Grants RO1 AI43955, HL50496, and HL078727.
